# Trick or treat?

**DOI:** 10.1186/1746-160X-3-22

**Published:** 2007-05-11

**Authors:** Murat Cehreli, Zafer Cehreli, Thomas Stamm, Ulrich Meyer, Hans-Peter Wiesmann

**Affiliations:** 1 CosmORAL Oral and Dental Health Polyclinics, Section of Prosthodontics, Ankara, Turkey; 2 Department of Pediatric Dentistry, Hacettepe University, Ankara, Turkey; 3 Department of Orthodontics, University of Muenster, Munster, Germany; 4 Clinic for Cranio-Maxillofacial Surgery, University of Düsseldorf, Düsseldorf, Germany; 5 Clinic for Cranio-Maxillofacial Surgery, University of Münster, Münster, Germany

## Abstract

The purpose of this article is to draw attention to current transgressions in scientific writing and to promote commitment to ethical standards and good science. All participants of any research project, particularly under interdiciplinary team approach, should not only play an active role on the management and carrying out of their study but also ensure that their study is not fraudulent. Manuscript fabrication, data and/or figure manupilation, piracy (plagiarism), sloppy research, and transgressions in authorship are reasons for loss of scientific value and records, retraction of articles, and application of a variety of sanctions.

"...His heart as far from fraud as heaven from earth."

*Shakespeare*, *The Two Gentlemen of Verona*

*Head & Face Medicine *was launched in 2005 as an open access "intra-interdiciplinary" journal with a vision to guide researchers of different fields to a particular target and to improve medical quality [1]. So far, the open peer-review process of *Head & Face Medicine *has been very efficient in creating a transparent and unanonymous communication between the author(s) and the reviewers. Because the identities of the reviewers are accessible, transgressions such as blocking the publication of a competitor or raking new ideas/methods from submissions, which are then rejected deliberately by licentious reviewers, are infrequent in the open peer-review process. Nonetheless, misconduct, fraud, and plagiarism is still unavoidable in scientific publishing, particularly in biomedicine, where the battle and commercial pay-offs are immense and many run for fame and fortune. As many developing countries and institutions strive for international recognition, institutional or even governmental financial rewards are offered to authors for high-profile papers [2]. Eventually, such treats trigger tendency toward trickery.

It is nice to be recognized as an innovative and distinguished scientist, but "only" through commitment in unhindered research undertaken for the "welfare of humanity" [3]. Hwang and Schøn [4,5] are among the most unfortunate impostors in the poignant history of fraud, not only because for being the victim of their own greed for eminence and lack of conscience, but also for being among the very few of captured, exposed to public, and condemned. Mainly published in high-impact journals, 0.02% (out of 400.000) fraudulent papers are believed to appear annually in journals covered by PubMed [6]. Fabrication, data/figure manupilation, replication, are among the transgressions cited for retraction of those impugned articles [6-8]. Nevertheless, it is tempting to speculate the extremely low number of transgressions detected thus far and vigilantly ask for the sheer truth of trickery. The Editors of *The Journal of Cell Biology *have claimed that up to 20% of accepted papers contained some questionable data, a rate that did not decrease since the journal instituted an editorial data-screening process [7]. The same rule applies to different fields of research. For example, 8% of articles published in orthopedics appear to contain transgressions [9]. One could, therefore, envisage that incidences of transgressions could presumably multiply and multiply without any detection in low-impact journals, and even surpass the limits of human prediction.

Another critical factor in scientific writing is authorship. With regard to its consequences on the scientific value of the study undertaken, this problem seems less important, but it is still widespread in universities and institutions. Authorship could be classified as coercion authorship, mutual support/admiration authorship, the gift authorship, the ghostwriter, and duplicate production authorship [10]. As the content of a study gets more complex, the need for allocation of roles and trust between participants in the research team, such as interdiciplinary team approach, becomes more important. The review process of any interdiciplinary study alone can neither catch the ghostwriter nor the gift author. Although it is also impossible to hunt the vampire (coercion author) and save the victims in a research team, if any, one of the prerequisites for having a paper published in *Head & Face Medicine *has been established as unambiguous justification of the contribution of each author in the submissions. Like many other journals, *Head & Face Medicine *also asks for competing interests (conflict of interest) from the authors in their submissions. The competing interests of the authors inculde financial, organizational, career and personal conflicts. Competing interests do not downgrade the scientific validity of the study, but exposes potential bias for involvement of the researcher(s) with any commercial product tested. A section relating to competing interests of the authors, therefore, appears in the guidelines for preparation of a manuscript for *Head & Face Medicine*.

We know that avoidance of scientific fraud is impossible solely by authorizing institutional or governmental boards to respond to allegations by performing sanctions. Education of research ethics and commitment to good science could drop the prevalence of deceitful papers. The Editorial Team of *Head & Face Medicine *sincerely hopes not only senior researchers but also junior researchers and students who are committed to scientific research will also be committed to current ethical standards and good science. Ethical standards raise the bar of scientific quality and trustworthiness. We strongly encourage authors to support our journal with novel "intra-interdiciplinary" studies.
